# Pausing the Fight Against Malaria to Combat the COVID-19 Pandemic in Africa: Is the Future of Malaria Bleak?

**DOI:** 10.3389/fmicb.2020.01476

**Published:** 2020-06-18

**Authors:** Nora Nganyewo Nghochuzie, Charles Ochieng' Olwal, Aniefiok John Udoakang, Lucas Naam-Kayagre Amenga-Etego, Alfred Amambua-Ngwa

**Affiliations:** ^1^West African Centre for Cell Biology of Infectious Pathogens (WACCBIP), University of Ghana, Accra, Ghana; ^2^Department of Biochemistry, Cell and Molecular Biology, College of Basic and Applied Sciences, University of Ghana, Accra, Ghana; ^3^Medical Research Council Unit The Gambia at LSHTM, Banjul, The Gambia; ^4^London School of Hygiene and Tropical Medicine, University of London, London, United Kingdom

**Keywords:** malaria, *plasmodium*, COVID-19, SARS-CoV-2, ACE2, coronaviruses

## Abstract

Malaria remains a major global health burden, killing hundreds of thousands annually, especially in sub-Saharan Africa. In 2019, a Phase IV Expanded Programme on Immunization (EPI)-linked malaria vaccine implementation was underway. However, in December 2019, a novel pneumonia condition termed coronavirus disease 2019 (COVID-19), caused by severe acute respiratory syndrome coronavirus 2 (SARS-CoV-2), with many clinical, epidemiological, and biological parallels to malaria, was reported in Wuhan, China. COVID-19 is spreading rapidly, and, as of the 3rd of June, 2020, more than 382,507 persons had died from COVID-19. Children under 5 years who suffer high malaria-attributable mortalities are largely asymptomatic for COVID-19. Considering that the malaria burden is highest in low-income tropical countries with little capacity to fund malaria control and eradication programs, the fight against malaria in these regions is likely to be hampered. Access to healthcare has generally been limited, while malaria interventions, such as seasonal malaria chemotherapy and distribution of insecticide-treated bed nets, have been suspended due to lockdowns. Likewise, the repurposing of antimalarials for treatment of COVID-19 shared symptoms and the shift in focus from the production of malaria rapid diagnostic tests (RDTs) to COVID-19 RDTs is a cause for concern in malaria-endemic regions. Children are less affected by the COVID-19 pandemic compared to the elderly. However, due to the fears of contracting SARS-CoV-2, the elderly who are worst affected by COVID-19 may not take children for malaria medication, resulting in high malaria-related mortalities among children. COVID-19 has disproportionately affected developed countries, threatening their donation capacity. These are likely to thwart malaria control efforts in low-income regions. Here, we present perspectives on the collateral impact of COVID-19 on malaria, especially in Africa.

## Introduction

Malaria, caused by infection with *Plasmodium* species parasites, remains a major health burden globally. In 2018, 228 million malaria cases and 405,000 deaths were reported worldwide, with sub-Saharan Africa bearing the greatest brunt (WHO, 2018). The global fight against malaria was at its peak with a Phase IV Expanded Programme on Immunization (EPI)-linked malaria vaccine implementation underway in Ghana, Malawi, and Kenya (WHO, 2020c).

In December 2019, unfortunately, a novel pneumonia condition termed coronavirus disease 2019 (COVID-19), caused by severe acute respiratory syndrome coronavirus 2 (SARS-CoV-2), with many epidemiological, clinical, and biological parallels to malaria, was reported in Wuhan, China (Hoffmann et al., 2020). Initial reports appeared local, but COVID-19 rapidly spread into a global pandemic, as declared by The World Health Organization (WHO) on the 11th of March, 2020. As of the 3rd of June, 2020, there were more than 6453,781 confirmed cases in 215 countries and territories, and fatalities exceeded 382,507 (https://www.worldometers.info/coronavirus/).

SARS-CoV-2 is the seventh member of the coronaviruses, a group of diverse, enveloped, positive-sense, single-stranded RNA viruses (Yan et al., 2020). SARS-CoV-2 most probably originated from bats given a 96.2% genome nucleotide sequence identity to a coronavirus from *Rhinophus affinis* bat (Zhou et al., 2020). COVID-19 is less severe in females compared to males but more pathogenic in older persons than youths (Cheng et al., 2020). Children under 5 years are largely asymptomatic for COVID-19, but they suffer high rates of malaria-attributable mortalities (WHO, 2014; Dhochak et al., 2020).

Considering that the malaria burden is highest in most low-income countries with little capacity to fund malaria control and eradication programs (Haakenstad et al., 2019), the fight against malaria in these regions is likely to be impacted negatively by the COVID-19 pandemic. COVID-19 at the time of writing this perspective has been disproportionately affecting the developed countries. Consequently, these countries that mostly fund malaria control and interventions may devote disease control resources inwards, and this could jeopardize resources for malaria control efforts in the low-income regions.

Here, we present our views on the potential collateral impact of COVID-19 on malaria and the likely implications of COVID-19 pandemic on malaria, especially in Africa.

## SARS-CoV-2 Receptor and Malaria Severity

SARS-CoV-2 uses angiotensin-converting enzyme 2 (ACE2) as a receptor for entry into the human cells (Yan et al., 2020), leading to the downregulation of ACE2 in infected persons (Sommerstein et al., 2020). ACE is characterized by a genetic deletion/insertion (D/I) polymorphism, which alters the concentration of ACE I and D allele, leading to reduced expression of ACE2. Low D allele frequencies have been reported in some countries affected by the pandemic, such as China and Korea (Delanghe et al., 2020). Regarding malaria, a study in India previously associated the D allele of ACE D/I polymorphism with malaria (Dhangadamajhi et al., 2010). Notably, the D allele of ACE D/I polymorphism increases angiotensin II. Another ACE2 polymorphism (C1173T substitution) also increases angiotensin II (Dhangadamajhi et al., 2010). Furthermore, analysis of persons of African descent reported several ACE1 and ACE2 polymorphisms. These polymorphisms increase angiotensin II, thereby conferring protection against severe malaria in children (Gallego-Delgado and Rodriguez, 2014). Angiotensin II has been shown to decrease *P. falciparum* invasion of human erythrocytes in a dose-dependent manner (Saraiva et al., 2011). Since geographical variations exist for ACE D/I polymorphism (Saab et al., 2007), determining the frequency and distribution of D allele and other polymorphisms in African populations is warranted to forecast the severity of infections with both pathogens toward defining appropriate intervention approaches and strategies to mitigate severe disease and deaths.

## Repurposing Antimalarials for Treatment of COVID-19

Artemisinin combination therapy (ACT) is the current antimalarial regime used in most endemic countries. However, emerging artemisinin resistance has been reported (Noedl et al., 2008; Dondorp et al., 2009). Recently, Madagascar reported a tonic of *Artemisia annua*, a plant that contains artemisinin, as a potential cure for SARS-CoV-2 (Finnan, 2020). The plant extract is likely to be officially or illicitely adopted for COVID-19 therapy by many countries in a desperate attempt to avert COVID-19-related deaths despite the WHO warning against its use without appropriate scientific approval. The WHO also discourages the use of the artemisia herb against malaria (WHO, 2012), though alternative medicine and pharmaceutical industries are likely to exploit the plant for SARS-CoV-2 treatment. Any widespread use of artemisia for SARS-CoV-2 infections will likely potentiate the development of ACT resistance, especially in Africa where malaria burden remains highest. This will spell doom for malaria elimination plans across African countries and other malaria-prone regions, as artemisinin monotherapy will drive resistance development to the main first-line malaria drugs, artemisinin-based combination therapy. Resistance to most of the combination partners in ACTs (Amodiaquine, Lumefantrine, mefloquine, and Sulphadoxine-pyrimethamine) has been well-demonstrated already (Sibley et al., 2001; Picot et al., 2009). Whereas, saving the world from COVID-19 using artemisia is a noble course, its ramifications on the malaria burden should therefore be of significant global health relevance. This calls for continuous enrichment of the antimalaria armamentarium with alternative drugs to avert eminent malaria catastrophe.

Moreover, antimalarials like chloroquine and hydroxychloroquine are being used for the management of SARS-CoV-2 against the advice of the WHO (Principi and Esposito, 2020). However, recently, a study suggested that chloroquine or hydroxychloroquine might be harmful in hospitalized COVID-19 patients (Funck-Brentano and Salem, 2020). Although the use of chloroquine against malaria parasites had been officially discontinued in most endemic areas owing to resistant parasites, recent studies have hinted at their possible reintroduction for malaria treatment (Mwanza et al., 2016; Bwire et al., 2020). The use of chloroquine and its derivatives in COVID-19 treatment is likely to facilitate the rebound of chloroquine-resistant malaria parasites. This will hinder the possible use of chloroquine and its derivatives in the fight against malaria in the future and thus further dampen the prospects of malaria elimination from Africa.

## Shifting Antigen (or Antibody)-Based Malaria Rapid diagnostic Tests (mRDTs) Production to COVID-19 RDTs

There is evidence that companies such as SD Bioline are shifting their mRDT production focus and repurposing the production pipelines to COVID-19 RDTs (WHO, 2020a). RDTs are the most widely used diagnostic tools for malaria. Therefore, the shortage of mRDTs as a direct result of these actions is eminent and will potentially jeopardize the WHO's test, treat, and track policy for malaria control. It is also feared that the shortage of mRDTs may result in presumptive treatment of malaria fever with associated cost implications and increased risk of antimalarial drug resistance. Overall, these actions (or inactions) might lead to an increase in malaria-attributable morbidity and mortality in the WHO Africa Region, which accounted for an estimated 93% of the global malaria cases and 94% of malaria deaths in 2018.

## Fighting COVID-19 at the Expense of Malaria: is the Risk Inevitable?

The world is fully focusing on COVID-19 at the expense of other endemic diseases, such as malaria, that also have high rates of mortality, especially in low-income countries. For instance, there was an outbreak of malaria in Zimbabwe, resulting in at least 131 deaths during the period of SARS-CoV-2 lockdown (Global development, 2020). Furthermore, Cameroon reported a substantial upsurge in malaria cases and deaths during the COVID-19 pandemic (Kindzeka, 2020). Reportedly, the malaria deaths in Zimbabwe and Cameroon were attributed to the shortage of malaria drugs and lack of access to medical facilities. This trend is likely to be replicated in other African and Asian countries with high malaria burden. Indeed, the WHO recently warned that the malaria mortalities in sub-Saharan Africa could double to 769,000 in 2020 following the disruptions of malaria control efforts by the COVID-19 pandemic (WHO, 2020d). In view of this, the WHO is calling for attention toward malaria interventions while responding to the pandemic to avoid the unintended consequences of SARS-CoV-2 on malaria in Africa (WHO, 2020b). One way to mitigate this loss of attention to malaria is to include both COVID-19 and malaria diagnoses in cases of fever. The expansion of COVID-19 serological surveys could also be complemented with survey for malaria to determine any patterns in co-transmission.

## Differential Susceptibility to COVID-19 and Malaria With Age

Most of the current evidence shows that SARS-CoV-2 is less pathogenic in young children, but contrastingly, young children are worst hit by malaria. The exact opposite is true for the adult population especially the elderly who are more likely to develop severe COVID-19 rather than malaria. Although the molecular basis of how COVID-19 could affect the malaria burden among children is not well-understood, the socio-economic and behavioral changes that come with the closure of national borders, population lockdowns, the concomitant collapse of small businesses, and financial stress imposed on families, particularly in rural populations, may indirectly affect household response to malaria and increase malaria burden in children. Indeed, SARS-CoV-2 has adversely affected people's health-seeking behavior. In general, people are hesitant to attend health care facilities due to fears of contracting COVID-19. This could affect the response of parents and caregivers in seeking remedial care for children with fevers that could be due to malaria. It is likely for example that grandparents who hitherto will take their grandchildren for medical care against malarial fever may hold back as they are more likely to die of COVID-19 infection and are advised to stay at home. These changes in health-seeking behavior may negatively impact malaria preventive measures and case management, thereby ultimately leading to an increase in malaria home management, which could, in turn, lead to more severe malaria and deaths from late reporting to health facilities.

## Shared Symptoms Between Malaria and COVID-19: Should We Worry?

The common clinical symptoms of SARS-CoV-2 infection, such as headaches, body aches, fatigue, and fever, are also applicable to malaria infections. Moreover, some malaria and SARS-CoV-2**-**infected individuals remain asymptomatic and undiagnosed and continuously spread the respective pathogens (Chanda-Kapata et al., 2020). The effect of *Plasmodium* species and SARS-CoV-2 coinfection on the immune response is not fully appreciated. SARS-CoV-2 and *P. falciparum* infections cause chronic inflammation (Ademolue et al., 2017; Fu et al., 2020), which could trigger cancers and other chronic diseases (Hattori and Ushijima, 2016). Further studies are therefore needed to understand the interactions between these pathogens, host immune responses, and disease sequelae in co-infected communities in Africa.

## Conclusions and Way Forward

SARS-CoV-2 and other infectious pathogens that may emerge later are likely to impact the global economy and health, in unmatched proportions, particularly for weak and vulnerable economies and public health systems in Africa. Increased continental disease surveillance and relevant research are required to understand the implications of the current COVID-19 pandemic on other endemic infectious diseases in Africa. The Africa Center for Disease Control must use its leverage within the African Union to encourage the allocation of funds by member states for disease surveillance, local scientific research, and interventions. We have highlighted the potential impact of SARS-CoV-2 infection on malaria, particularly in Africa. Repurposing of antimalarials and the development of diagnostics for COVID-19 must be handled in a manner that does not jeopardize the gains made in controlling malaria and other endemic diseases. More concerted efforts are required to concurrently monitor the two diseases. Particularly, malaria diagnosis could be coupled to the COVID-19 screening and testing of suspected or confirmed COVID-19 patients to avoid misdiagnosis and enable easy management. Neglecting malaria in favor of COVID-19 could prove catastrophic for global health, particularly in Africa.

## Author Contributions

NN and CO conceived and prepared the first draft of the manuscript. AU, LA-E, and AA-N critically reviewed the draft. All the authors approved the final version of the manuscript.

## Conflict of Interest

The authors declare that the research was conducted in the absence of any commercial or financial relationships that could be construed as a potential conflict of interest.
